# Successful treatment of acute promyelocytic leukemia in a patient with prosthetic heart valves: a case report and review of the literature

**DOI:** 10.1186/s13256-021-02825-2

**Published:** 2021-05-04

**Authors:** Shirin Haghighat, Alireza Rezvani, Maral Mokhtari

**Affiliations:** 1 Department of Hematology and Medical Oncology, Shiraz University of Medical Sciences–Namazi Hospital, Zand Avenue, Shiraz, Iran; 2 Department of Hematology and Medical Oncology, Hematology Research Center, Shiraz University of Medical Sciences, Shiraz, Iran; 3 Faghihi Hospital–Shiraz University of Medical Sciences, Shiraz, Iran

**Keywords:** Acute promyelocytic leukemia, DIC, Thrombosis, Treatment, Case report

## Abstract

**Background:**

Although life-threatening hemorrhage is a usual manifestation of acute promyelocytic leukemia (APL), thrombotic events seem to be more common in APL compared to other subtypes of acute leukemia. The treatment and prophylaxis of thrombosis are controversial due to the high risk of bleeding caused by disseminated intravascular coagulation (DIC) and thrombocytopenia. To the best of our knowledge we report the first case of APL in a patient with prosthetic heart valves successfully treated with a combination of all-trans-retinoic acid (ATRA) and arsenic trioxide (ATO). We hope this case report helps clinicians to manage different spectra of coagulopathy in APL successfully.

**Case presentation:**

A 38-year-old Asian man presented with diagnosis of APL confirmed by bone marrow biopsy. He was on warfarin due to prosthetic mitral and aortic valves. He was at risk of both hemorrhagic events due to DIC and life-threatening valve thrombosis. Our management regimen included unfractionated heparin adjusted according to the platelet count to prevent both valve thrombosis and bleeding events. The patient tolerated treatment well without any hemorrhagic or thrombotic events, and complete molecular remission was achieved by ATRA and ATO without the need for chemotherapeutic agents.

**Conclusion:**

Although this case is exceptional, a precise evaluation may be needed to select the appropriate dose and type of anticoagulant to treat a patient with APL.

## Introduction

Acute promyelocytic leukemia (APL) is a specified subtype of acute myelogenous leukemia (AML) characterized by thrombo-hemorrhagic syndrome [1]. The disease is diagnosed based on bone marrow infiltration by leukemic abnormal promyelocytes [2]. APL is associated with the presence of hallmark cytogenetic abnormality t(15;17) which results from the fusion of the retinoic acid alpha (RARalpha) and PML genes [3]. While bleeding diathesis is the predominant manifestation of the coagulopathy that frequently occurs in patients with APL and the main cause of early mortality in these patients, clotting activation and subsequent thrombotic events are life-threatening complications in APL and sometimes underestimated in this disease [4]. An upgraded knowledge of these two spectrums of coagulation abnormalities, i.e. haemorrhage and thrombosis, is necessary to improve the management of APL. Here we report a 37-year-old man with prosthetic mitral and aortic valves who presented with APL and coagulopathy.

## Case presentation

**Fig. 1:**
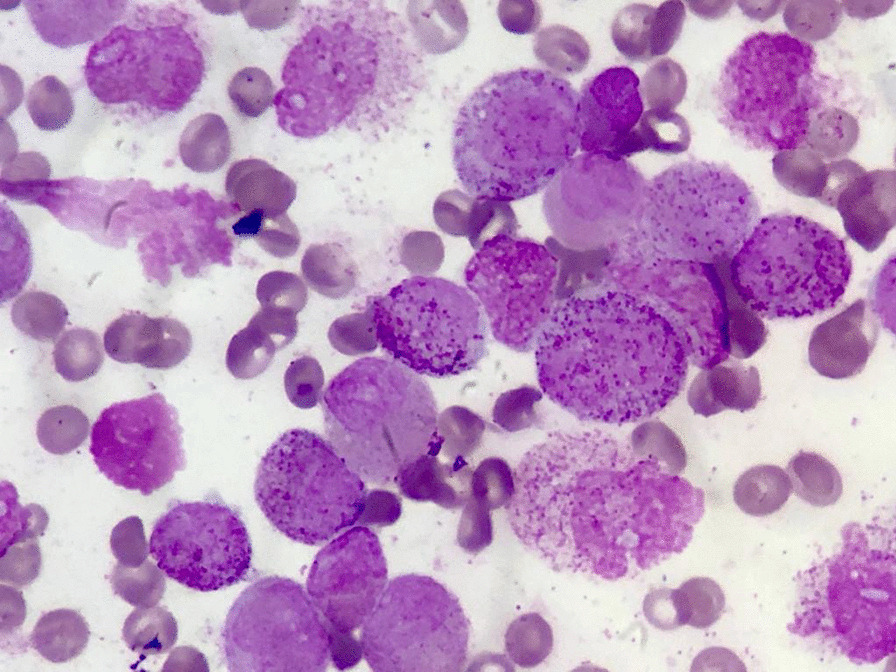
Bone marrow aspirate shows numerous highly granular promyelocytes, characteristic of acute promyelocytic leukemia, Wright stain  × oil immersion (1000)

A 38-year-old married Asian man was admitted to the Emergency Department of a referral hospital due to a few days of fever, chills and cough. He also complained of malaise and tiring easily, unusual for a young man. Physical examination showed pallor in the absence of lymphadenopathy or hepatosplenomegaly. He was febrile with temperature of 38.3 °C and had only patchy ecchymosis on his extremities. Respiratory rate was 20 breaths per minute and oxygen saturation by pulse oximetry was 92% in room air. The results of other aspects of the physical examination were unremarkable except for a loud opening click related to the prosthetic valves. He had undergone prosthetic mitral and aortic valve replacement 16 years prior to the current presentation, for which he was on the oral anticoagulant warfarin. Initial laboratory values were remarkable for white blood cell count (0.7 × 10^9^/L), platelet count (58 × 10^9^/L), haemoglobin (6.8 g/dL), prothrombin time (24.1 s), international normalized ratio (INR; 2.95), partial thromboplastin time (PTT; 34.5 s), lactate dehydrogenase (5530 U/L), D-dimer (164 ng/mL) and fibrinogen level (106 mg/dL). Peripheral smear did not show abnormal cells. Computerized tomography of the chest revealed a consolidation in the upper lobe of the right lung and evidence of patchy ground glass opacities in the lower lobe of the right lung. Treatment with broad-spectrum antibiotics was initiated immediately, which resulted in a good response followed by a return to normal temperature after 2 days. Bone marrow aspiration showed a massive infiltration of abnormal promyelocytes consistent with APL (Fig. 1). Flow cytometry revealed a strong positivity for CD117, CD33, CD45 and negativity for HLADR, CD5, CD7, CD20 and CD19. Quantitative restriction transcription-polymerase chain reaction (PCR) showed positivity for t(15;17). Transthoracic echocardiography showed normal function of the prosthetic mitral and aortic valves and a left ventricular ejection fraction of 50%.

We considered the diagnosis of low-risk APL and immediately started the patient on all-trans-retinoic acid (ATRA) at 45 mg/m^2^ and arsenic trioxide (ATO) at the standard dose of 0.15 mg/kg. Treatment with the oral anticoagulant was discontinued and changed to unfractionated heparin with dose adjustment according to the platelet count and activated PTT (aPTT). Because the baseline aPTT was in the normal range, we adjusted the dose of heparin to achieve twice normal-level aPTT. We tried to keep the platelet count above 30,000/µL with a daily infusion of single-donor apheresis platelets. Although the disseminated intravascular coagulation (DIC) profile showed an increase in PT and D-dimer and a decrease in fibrinogen level, the patient did not receive fresh frozen plasma or cryoprecipitate due to lack of any evidence of active bleeding and the possible risk of valve thrombosis. The DIC profile was successfully normalized with the initiation of ATRA and ATO. The platelet count was monitored daily, with plans to transfuse platelets to a level of 50 × 10^3^/µL. On day 4 of treatment, the platelet count dropped below 20 × 10^3^/µL, and heparin was subsequently discontinued despite the concern about the risk of valve thrombosis. At the same time, the patient also developed high-grade fever again, and blood culture ultimately revealed bacteraemia by methicillin-resistant* Staphylococcus aureus*. Antibiotics were changed based on the results of the antibiogram test and two-dimensional transthoracic echocardiography was performed, which showed no evidence of endocarditis nor valve thrombosis. Heparin was restarted cautiously as the patient carried a high bleeding risk. Following recovery of the peripheral blood cell count on day 20 of therapy with ATRA and ATO, heparin was changed to warfarin and the patient was discharged from hospital. Bone marrow biopsy also demonstrated a complete morphological response. He was subsequently placed on consolidation therapy with ATRA and ATO. The patient is currently in molecular complete remission at 16 months from diagnosis.

## Discussion

In patients with mechanical valve replacement, life-long oral anticoagulant therapy with vitamin K antagonists and an INR in the target range are crucial [5]. Prosthetic valve thrombosis (PVT) is one of the main causes of valve dysfunction and can be a life-threatening event [6]. The most common causes of PVT are insufficient anticoagulation, hypercoagulable states, such as pregnancy, atrial fibrillation and left ventricular dysfunction [7]. In addition, thromboembolic events may be a common complication among patients with APL through several mechanisms, such as prolonged immobility and the administration of ATRA alone or in combination with antifibrinolytic agents, genetic predisposition or as a consequence of differentiation syndrome [8]. The thrombotic events seem to be more common in APL than in other types of acute leukemia, with a reported prevalence rate ranging from 2 to 10–15% [9]. One of the potential mechanisms involved in the pathogenesis of thrombosis in APL includes the interaction of leukemic cells with endothelial cells and activation of the clotting system and subsequent thrombus formation [10]. There is an ongoing debate on the prophylaxis and treatment of thromboembolic events in patients with APL due to the high risk of bleeding resulting from DIC and thrombocytopenia [1]. Although there is some evidence that heparin can be beneficial in the setting of DIC, the use of heparin is not recommended in patients with APL due to the lack of evidence from clinical studies and high risk of bleeding [11]. To the best of our knowledge, no randomized controlled trial data are available on the dose and type of anticoagulant for the treatment of patients with APL.

Also, there has not been any specific recommendation on the safety and permissive duration of withholding anticoagulants in patients with mechanical valve replacement. Krittalak *et al.* evaluated 26 patients with a mechanical heart valve who were hospitalized with intracranial haemorrhage (ICH) and found that withholding anticoagulation treatment for < 7 days was associated with a low risk of thromboembolic events [12]. Results from other studies suggest restarting anticoagulation treatment not earlier than 2 weeks after ICH in patients with a prosthetic heart valve and reserving earlier restarting of anticoagulant treatment only for patients at high risk of thromboembolic events [13, 14].

Given the complex situation of our patient in association with the risk of prosthetic valve thrombosis in a context of bleeding tendency (disseminated intravascular coagulation and thrombocytopenia), choosing the type and dose of anticoagulation was challenging. Ahrari *et al.* conducted a systematic review to determine the choice, dose, duration, efficacy and safety of anticoagulation in patients with acute leukaemia and thrombosis [15]. Conventional heparin and low-molecular-weight heparin were the most commonly used anticoagulants in all studies. These authors revealed that dose adjustment of anticoagulant was made according to the severity of thrombocytopenia [15]. Our patient was placed on conventional heparin instead of warfarin due to the shorter half-life of heparin and the availability of an antidote to reverse the effect of anticoagulation rapidly following any sign of bleeding. Also, we adjusted the dose of heparin according to the platelet count to reduce the risk of bleeding. Additionally, the combination of ATRA and arsenic without chemotherapy was not only effective in inducing complete remission in our patient but it also reduced the risk of prolonged severe thrombocytopenia caused by chemotherapy.

## Conclusion

We managed a complex patient with prosthetic valve and APL successfully with a modified dose of heparin to avoid both bleeding and valve thrombosis. A precise evaluation must be done to select the appropriate dose and type of anticoagulant in a patient with APL.

## Data Availability

No datasets were generated or analysed during this study.

## Abbreviations

APL: Acute promyelocytic leukemia
AML: Acute myelogenous leukemia
WBC: White blood cells
INR: International normalized ratio
ATRA: All-trans-retinoic acid
ATO: Arsenic
FFP: Fresh frozen plasma
DIC: Disseminated intravascular coagulation
VKA: Vitamin K antagonist
PVT: Prosthetic valve thrombosis
ICH: Intracranial hemorrhage
LMWH: Low molecular weight heparin
